# The Path to Conserved Extended Haplotypes: Megabase-Length Haplotypes at High Population Frequency

**DOI:** 10.3389/fgene.2021.716603

**Published:** 2021-08-06

**Authors:** Chester A. Alper

**Affiliations:** ^1^Program in Cellular and Molecular Medicine, Boston Children’s Hospital, Boston, MA, United States; ^2^Department of Pediatrics, Harvard Medical School, Boston, MA, United States

**Keywords:** complement deficiency, complotype, haplotype, HLA, MHC, pedigree, polymorphism

## Abstract

This minireview describes the history of the conceptual development of conserved extended haplotypes (CEHs): megabase-length haplotypes that exist at high (≥0.5%) population frequency. My career began in internal medicine, shifted to pediatrics, and clinical practice changed to research. My research interest was initially in hematology: on plasma proteins, their metabolism, synthesis, and function. This narrowed to a focus on proteins of the human complement system, their role in immunity and their genetics, beginning with polymorphism and deficiency of C3. My group identified genetic polymorphisms and/or inherited deficiencies of C2, C4, C6, and C8. After defining glycine-rich beta glycoprotein as factor B (Bf) in the properdin system, we found that the genes for Bf (*CFB*), C2, C4A, and C4B were inherited as a single haplotypic unit which we named the “complotype.” Complotypes are located within the major histocompatibility complex (MHC) between *HLA-B* and *HLA-DRB1* and are designated (in arbitrary order) by their *CFB*, *C2*, *C4A*, and *C4B* types. Pedigree analysis revealed long stretches (several megabases) of apparently fixed DNA within the MHC that we referred to as “extended haplotypes” (later as “CEHs”). About 10 to 12 common CEHs constitute at least 25 – 30% of MHC haplotypes among European Caucasian populations. These CEHs contain virtually all the most common markers of MHC-associated diseases. In the case of type 1 diabetes, we have proposed a purely genetic and epigenetic model (with a small number of Mendelian recessive disease genes) that explains all the puzzling features of the disease, including its rising incidence.

## Introduction

During the period from 1981 to 1983, my research group published findings (Awdeh et al., 1981, 1983; Alper et al., 1982), using family-based (“pedigree”) data, showing the existence of high-frequency population-level megabase (Mb)-length conserved extended haplotype (CEH) variants (“CEHs”) in the human major histocompatibility complex (MHC), what we then termed MHC “extended haplotypes.” In the late 1980s, a research group in Perth led by Roger Dawkins confirmed (Tokunaga et al., 1989), using both serological and DNA-based methods primarily on immortalized cell lines from unrelated subjects homozygous for at least some portions of the MHC (Kay et al., 1988; Tokunaga et al., 1988), that what they had previously termed MHC “supratypes” (Dawkins et al., 1983) were MHC “ancestral haplotypes” (AHs; Degli-Esposti et al., 1992; Dawkins et al., 1999) and that these were essentially the equivalent of CEHs. Additionally, data had been published several years prior to our own work that, as we cited in our papers of the early 1980s, provided further evidence for the existence of CEHs in the French population (Dausset et al., 1978). Thus, CEHs and AHs are synonyms for Mb-length conserved polymorphic sequence variants (each unique sequence having multiple copies in a population among otherwise apparently unrelated pedigrees) that exist at far higher than expected population-level frequencies (based on their length and the polymorphic allele frequencies of their component marker loci).

I cover several topics related to the discovery and relevance of MHC complotypes and CEHs. The primary focus is the work that led to the discovery of MHC CEHs: both my own laboratory’s clinical family-based complement polymorphism studies and contemporaneous hypotheses arising from family-based and population-level HLA antigen polymorphism studies. The former directly led to my group’s discovery, through serological typing of variants for each of four MHC-linked complement gene products, of the “complotype.” The complotype genes turned out to cover an approximately 140 kilobase (kb) region containing, along with other genes, four complement genes in the central region (between the HLA class I and class II regions) of the human MHC. By 1981, our own HLA and complement typing data supported the complotype concept to extend the length of the “fixed” haplotypes to at least the region from *HLA-B* to *HLA-DRB1* (then, HLA-DR) covering over 1 cM (now known to be approximately 1.24 Mb in the human genome reference sequence).

The remaining sections review: (a) technical advancements in the definition of HLA, complement and other MHC-associated gene alleles as well as advancements in our understanding of the core MHC genomic architecture; (b) CEH extension to most of the remaining MHC region; (c) identification of ethnic differences in MHC CEH distributions; (d) insights into MHC-associated genetic diseases achieved through the knowledge of and use of pedigree-based MHC haplotypes; and (e) the development of new genetic disease models as a consequence of considering the existence of CEHs.

## From Family-Based Clinical Hematology to Serological Immunogenetics

Throughout my career I have followed my curiosity. This has resulted in multiple major shifts, including from internal medicine to pediatrics, from patient care to research, from hematology to immunology and from immunology to genetics. After clinical training, 2 years of general medical practice and board certification in internal medicine, my interest in academic research began in 1959 in Albert Coons’ laboratory at Harvard Medical School. There, I learned basic protein analytical techniques, and I applied for and received a U.S. Public Health Service fellowship to study clinical hematology with Jan Waldenström and Carl-Bertil Laurell at the University of Lund in Malmö, Sweden. In the mid-1940s, Waldenström had described an immunoglobulin-M-producing hematologic neoplastic disorder which bears his name (Waldenström, 1958), and Laurell had just described alpha-1 antitrypsin deficiency (Laurell and Eriksson, 1963) and its resulting chronic obstructive pulmonary disease. So began my research odyssey. Under their superb guidance, my first studies were of the metabolism of immunoglobulins (Alper et al., 1963).

After returning to Boston, I joined Frank Gardner’s Hematology Division at what was then the Peter Bent Brigham Hospital. I was involved in studies of the synthesis and metabolism of plasma proteins such as haptoglobin, fibrinogen, immunoglobulins (Alper et al., 1965) and beta1c globulin (Alper et al., 1966). Studies of the latter showed it to be the third component of complement, C3 (Klemperer et al., 1965), and the liver was its primary (Alper et al., 1969a) although not only (Einstein et al., 1977; Carroll, 1998) site of synthesis.

On moving to Boston Children’s Hospital under Charles Janeway, I set up a diagnostic serum protein analysis service (modeled after Laurell’s clinical laboratory service at Malmö General Hospital) at the Blood Grouping Laboratory [then headed by Louis K. Diamond (Alper et al., 2002)]. This is where I began family-based (“pedigree”) studies to evaluate complement protein polymorphisms. My interest in complement was initiated by studies of the human C3 complement protein *in vivo*, which led to the identification (with Fred Rosen) of the metabolism and early complement activation cascade immune function of C3 (Alper and Rosen, 1967).

We then began the methodical analysis of C3 genetic polymorphism (Alper and Propp, 1968) and its inherited deficiency (Alper et al., 1969b). My interest in the C3 genetic polymorphism led to the development, with Myron Johnson, of the technique of immunofixation electrophoresis (Alper and Johnson, 1969). This greatly facilitated the detection of genetic polymorphisms for a wide variety of proteins.

One of the plasma proteins analyzed by this technique was glycine-rich beta glycoprotein (Boenisch and Alper, 1970), and we showed it to be highly polymorphic with two common genetic variants (Alper et al., 1972). Further complement protein genetics studies demonstrated polymorphisms in C6 (Alper et al., 1975), C2 (Alper, 1976), C8 (Raum et al., 1979), and C4 (Awdeh et al., 1979). It was the demonstration (with Ira Goodkofsky and Irwin Lepow) that glycine-rich beta glycoprotein is Factor B (Bf, the product of what is now the named gene *CFB*) of the alternative complement pathway (Alper et al., 1973) that connected our interests in serum proteins, immunology, and genetics.

Independent of my group, during the late 1960s and early 1970s, two of my future collaborators also were working on new techniques to study immune-related protein variants. Zuheir Awdeh, then working at the National Institute for Medical Research in London, described a new method of isoelectric focusing in polyacrylamide gels (Awdeh et al., 1968). After several years in London working on protein separations, often with serum proteins, he moved first to the American University in Beirut and then, in the late 1970s, he joined my group in Boston. Meanwhile, Edmond Yunis, who had published biomedical studies for years prior, trained with D. Bernard Amos at Duke University in HLA typing in 1967, immediately set up an HLA typing laboratory at the University of Minnesota and published work independently and, separately, with both Robert Good (Yunis et al., 1967) and Amos (Amos and Yunis, 1969; Yunis and Amos, 1971). In the late 1970s, Edmond was recruited to Harvard Medical School and, separately, to manage the American Red Cross typing facility in Boston. He and I began collaborating soon thereafter.

## From Complement Immunogenetics to the Human MHC Complotype

In 1974, Fred Allen, Jr., of the New York Blood Center, published a report linking Bf to HLA (then termed “HL-A”) in humans (Allen, 1974), and we, in 1975, showed that Bf was linked to the MHC in the rhesus macaque (Ziegler et al., 1975). Allen and associates at The Rockefeller University also provided evidence linking C2 to HLA (Fu et al., 1974). Other groups independently, in 1975 and 1976, discovered close linkage between HLA markers in the human MHC and, separately, both with Bf and C2. My group’s demonstration of structural genetic polymorphism in C2 in family studies as well as genetic polymorphisms of other complement proteins in the same families led to our discovery of the close linkage between C2 and Bf (Alper, 1976). Thus, it was becoming clear that at least two complement genes were likely both closely linked to one another as well as to other human MHC markers.

Two years later, Jean Dausset’s group published on linkage disequilibrium (LD) between HLA-A, -B, -DR, Bf, and C2 (among other loci) in studies of 53 French families (Dausset et al., 1978). Although two-locus (Mattiuz et al., 1970) and three-locus (Piazza, 1975) LD within the human MHC region had been described for almost a decade using unrelated subject data, Dausset’s 1978 publication was the first using pedigree-based multi-locus LD analysis of both HLA and complement gene markers. They found multi-locus LD mostly between various MHC segments (one of which was from HLA-C to -DR). However, they suggested that long-range MHC haplotypes in strong disequilibrium were “relatively limited.” It is unclear whether the relatively primitive typing of the time, the sample size, the lack of C4 typing and/or the LD methodology they employed led to their conclusion of only “limited” LD throughout the MHC.

Soon thereafter, we found that there was both close linkage between these loci and C4 (Raum et al., 1980) and that human C4 could be viewed as two distinct genetic loci–C4A and C4B (Awdeh and Alper, 1980). These findings led us to describe the MHC BF-C2-C4A-C4B complement haplotypes as “complotypes” (Alper et al., 1983). We noted that we had seen no recombinants within this region after analyzing hundreds of meioses.

## Extending the Regional Complotype to Long-Range MHC CEHs (Family Studies)

Immediately upon developing the MHC complotype concept and prior to its publication, we published our hypothesis of and evidence for long-range MHC haplotypes containing complotypes in relation to HLA-A, HLA-B, and HLA-DR (Alper et al., 1982). Our work revealed “extended haplotypes” [Awdeh et al., 1983; later termed “conserved extended haplotypes” (Alper et al., 1992); and then abbreviated as CEHs (Yunis et al., 2003)]. Work from the 1980s to the 2000s extended the extent of “fixity” (sequence identity or near identity of population haplotype variants sharing the same major MHC markers of a single CEH) of CEHs from *HLA-A* to *HLA-DQB1*–a total distance of over 2 cM (now known to be approximately 2.72 Mb) and, to some extent from *HLA-A* to *HLA-DPB1* (3.15 Mb). CEH allele definition and haplotype extension beyond the *HLA-B* to *HLA-DRB1* region has progressed over the last nearly 40 years and is covered in the next section.

First, I wish to highlight the region from *HLA-C* to *HLA-DQB1* [a 1.4 Mb portion (40%), skewed toward the centromeric end, of the 3.48 Mb “classical” MHC region stretching from *ZFP57* to *HCG24* (Horton et al., 2004)] in terms of the population frequencies of CEHs and provide the briefest of summaries as to how those distributions vary between human populations. There are about 10–12 common (>1%) CEHs among European Caucasians (Alper et al., 1992; Yunis et al., 2003; Szilágyi et al., 2010). Such CEHs have a combined frequency of 25–30% in this population with specific CEHs varying in frequency among different European subpopulations (Szilágyi et al., 2010). Two reviews previously documented just a few of the CEHs in both Caucasian and non-Caucasian populations (Dawkins et al., 1999; Yunis et al., 2003).

Ethnic population allele variation within the MHC has been known since the beginning of HLA (Ceppellini et al., 1965) and complement (Alper et al., 1972) typing. Although most of my work has focused on European Caucasians, we began analyzing CEH population variation explicitly in the late 1980s by studying the salt-wasting disease congenital adrenal hyperplasia in ethnically admixed families from Venezuela (Layrisse et al., 1987). Our other early studies on CEH variants in populations other than dominant European Caucasian ethnicities include Ahmed et al., 1990 – among Ashkenazi Jews; Fraser et al., 1990 and Fraser et al., 1991 – among individuals of African and African-American descent; and Delgado et al., 1996 – among South Asian individuals from New Delhi and Ahmedabad. Both then (e.g., Kay et al., 1988) and more recently, other groups have reported on both previously and newly identified CEHs and their frequencies in families from Mexico (Zúñiga et al., 2013), Japan (Morishima et al., 2010; Ikeda et al., 2015) and Nigeria (Testi et al., 2015) – just to name a few. CEH variants and their relative frequencies vary widely, largely due to historical population admixture. Pedigree-analyzed MHC CEH population variation analyses have often been secondary to medical studies, and much further work remains to be conducted in non-European Caucasian populations to explore the full-range of human diversity within the MHC.

## MHC Allele Definition and CEH “Fixity” Analyses

Two excellent reviews on the evolving “map” of the human MHC (Horton et al., 2004) and HLA nomenclature (Hurley, 2021) provide the necessary context for understanding how the precision with which individual CEH variants have been defined has changed over the past 35 years. Not only have gene names changed (both before and after 2004), but the technology for identifying component alleles has switched from mostly serological to, starting in the early 1980s, DNA-based typing –the latter with rapidly increasing detail as DNA sequencing technology has advanced.

In the mid-1980s, purely DNA-based genetic allele analysis began its ascendance after MHC chromosomal mapping was achieved (e.g., the complotype region: Carroll et al., 1984; Carroll et al., 1985), and this led to further insights. Thus, available MHC complotype polymorphisms expanded (Schneider, 1990), and attempts were made by us (Whitehead et al., 1988; Truedsson et al., 1993; Simon et al., 1997) to correlate protein-based complotypes with DNA-based variants and by others to create a DNA-based nomenclature associated with MHC CEHs (Yu, 1998; Bánlaki et al., 2012; Sekar et al., 2016). Unfortunately, this region’s duplication and deletion complexity continues to make it extremely difficult to DNA sequence the central MHC even using consanguineous cell lines (Horton et al., 2008).

Some of our own work providing increasing allelic detail and evidence for fixity (or sub-type fixity) of various MHC CEH variants or their regional “block” components include: Kruskall et al., 1987; Whitehead et al., 1988; Egea et al., 1991; Yunis et al., 1993, 2003; Garcia-Merino et al., 1996; Turbay et al., 1997; Clavijo et al., 1998; Pinto et al., 2004; Bilbao et al., 2006; Romero et al., 2007; Larsen et al., 2014; Vadva et al., 2019. Other groups have also contributed to the further characterization of CEH/AH allelic definition and fixity. A small selection of these includes Zhang et al., 1990; Jongeneel et al., 1991; Degli-Esposti et al., 1992, 1995; Tay et al., 1997; Dawkins et al., 1999; Stewart et al., 2004; Aly et al., 2006; Smith et al., 2006; Traherne et al., 2006; Morishima et al., 2010; Kulski et al., 2011, 2021; Baschal et al., 2012; Askar et al., 2013; Lam et al., 2015.

## Studying Genetic Traits and a New Genetic Model Based on the CEH Concept

To study complex genetic disease, most geneticists use a “case vs. control” study design of unrelated subjects who are, putatively, demographically similar other than for disease status. Individual loci–typically, single nucleotide polymorphisms (SNPs) or, in the human MHC, individual HLA loci–are tested in such a design to localize the most likely marker(s) associated with genetic traits and diseases (Bush and Moore, 2012). In the case of type 1 diabetes (T1D), a very large number of genes throughout the genome exhibit small but significant differences in frequency in patients as compared to controls (Barrett et al., 2009; Reddy et al., 2011; Bakay et al., 2019). Genes marked by these SNPs are said to increase the “risk” of having the disease. In our view, these SNPs mostly mark different Caucasian subpopulations (Awdeh et al., 2006; Alper et al., 2019), and haplotype-based studies provide a much more complex but realistic source for testing genetic disease associations.

Our work has focused on a “disease vs. family control” haplotype study design using families (Fleischnick et al., 1983; Raum et al., 1984; Alper et al., 1987, 2000, 2006b; Hauser et al., 1989; Ahmed et al., 1993; Larsen and Alper, 2004; Alper and Larsen, 2015, 2017; Vadva et al., 2019). As recently noted, family study had always been a useful tool for studying human disease genetics (Bodmer, 2019). Case-control design is primarily used to save costs and time (i.e., not having to find willing family participants). Human genetics has moved away from family-based haplotype studies to unrelated subject genotype studies. In our view, preferable study methods include the disease-family control haplotype design or a design comparing specific haplotype homozygotes, heterozygotes, and non-carriers (Alper et al., 2000). This latter prospective method or one utilizing monozygotic twins (Alper et al., 2006a) can also be used to study incomplete penetrance of complex genetic traits (Alper and Awdeh, 2000).

Based on these considerations and our own findings in family studies, we have proposed a stochastic epigenetic Mendelian oligogenic (SEMO) model for T1D (Alper et al., 2019). We posit a small number (2 to 5) of (relatively) unlinked Mendelian recessive genes required for disease susceptibility. We attribute epigenetic alteration of one of the genes causing incomplete penetrance as determined by the approximately 50% rate of T1D concordance both in monozygotic twins of patients and in the offspring of two parents with T1D (Rjasanowski et al., 2003) as well as the onset of disease in late childhood. The SEMO model explains the rising incidence of T1D by noting that past selection against this life-threatening disease could be achieved through the reduction in frequency of any of the causal genes (Awdeh et al., 2006). If parents are from subpopulations that selected against different causal genes, the risk of complete disease susceptibility in the offspring will be higher than that of either parent. In support of this explanation is the observation that grandparental subpopulation mixing in T1D families at 54% is twice that of control families (27%) (Awdeh et al., 2006).

## Discussion

This review is my account of the discovery and implications of a little understood feature of the human MHC: the CEH. I summarized the main reports leading to demonstration of the existence of multiple CEH variants and several key reports investigating details of their structure and usage in the ensuing four decades. Also highlighted were several critical steps and methodologies that led to the CEH concept. Several features of CEHs yet to be determined were also described. CEHs may exist elsewhere in the human genome and likely those of at least some other diploid species, but further studies are required to delineate the extent to which each is true. This review could not cover all aspects of CEHs, but I attempted to focus on major developments. Many questions and a great deal of unexplored territory remains.

## Author Contributions

The author confirms being the sole contributor of this work and has approved it for publication.

## Conflict of Interest

The author declares that the research was conducted in the absence of any commercial or financial relationships that could be construed as a potential conflict of interest.

## Publisher’s Note

All claims expressed in this article are solely those of the authors and do not necessarily represent those of their affiliated organizations, or those of the publisher, the editors and the reviewers. Any product that may be evaluated in this article, or claim that may be made by its manufacturer, is not guaranteed or endorsed by the publisher.
